# How Tone, Intonation and Emotion Shape the Development of Infants’ Fundamental Frequency Perception

**DOI:** 10.3389/fpsyg.2022.906848

**Published:** 2022-06-03

**Authors:** Liquan Liu, Antonia Götz, Pernelle Lorette, Michael D. Tyler

**Affiliations:** ^1^MARCS Institute for Brain, Behaviour and Development, Western Sydney University, Penrith, NSW, Australia; ^2^Center for Multilingualism in Society Across the Lifespan, University of Oslo, Oslo, Norway; ^3^Australian Research Council Centre of Excellence for the Dynamics of Language, Canberra, ACT, Australia; ^4^Department of Linguistics, University of Potsdam, Potsdam, Germany; ^5^Department of English Linguistics, University of Mannheim, Mannheim, Germany

**Keywords:** lexical tone, intonation, Prosody, phonological theory, sensory processing, cognitive processing, cross-linguistic transfer, emotional tone

## Abstract

Fundamental frequency (*ƒ*_0_), perceived as pitch, is the first and arguably most salient auditory component humans are exposed to since the beginning of life. It carries multiple linguistic (e.g., word meaning) and paralinguistic (e.g., speakers’ emotion) functions in speech and communication. The mappings between these functions and *ƒ*_0_ features vary within a language and differ cross-linguistically. For instance, a rising pitch can be perceived as a question in English but a lexical tone in Mandarin. Such variations mean that infants must learn the specific mappings based on their respective linguistic and social environments. To date, canonical theoretical frameworks and most empirical studies do not view or consider the multi-functionality of *ƒ*_0_, but typically focus on individual functions. More importantly, despite the eventual mastery of *ƒ*_0_ in communication, it is unclear how infants learn to decompose and recognize these overlapping functions carried by *ƒ*_0_. In this paper, we review the symbioses and synergies of the lexical, intonational, and emotional functions that can be carried by *ƒ*_0_ and are being acquired throughout infancy. On the basis of our review, we put forward the Learnability Hypothesis that infants decompose and acquire multiple *ƒ*_0_ functions through native/environmental experiences. Under this hypothesis, we propose representative cases such as the synergy scenario, where infants use visual cues to disambiguate and decompose the different *ƒ*_0_ functions. Further, viable ways to test the scenarios derived from this hypothesis are suggested across auditory and visual modalities. Discovering how infants learn to master the diverse functions carried by *ƒ*_0_ can increase our understanding of linguistic systems, auditory processing and communication functions.

## Introduction

From the beginning of life, humans are exposed to the fundamental frequency (*f*_0_; Titze et al., 2015). The *f*_0_ carries a wide range of information. This includes linguistic (e.g., lexical tone), paralinguistic (e.g., speaker intent, emotion, Crystal and Quirk, 1964; Gussenhoven, 2002), and extralinguistic information (e.g., melody, Johnson, 1990; He et al., 2007). While some crucial communicative functions carried by *f*_0_ appear to be universal, such as intonation (Best, 2019), others can vary across the world’s languages (e.g., signalling grammatical information; Hyman, 2011, 2016; Remijsen, 2016). For example, a syllable /ja/ with a rising *f*_0_ can be recognised as an attention getter for a Dutch speaker, but as the word “tooth” for a speaker of Mandarin. Thus, to acquire the language of their environment, infants are faced with a complex task. They must learn to disambiguate, decompose, recognise, and learn the patterns of *f*_0_ variability that apply to different linguistic, paralinguistic, and non-linguistic domains.

It is impressive that infants process different sources of speech information and eventually learn to disentangle functions of *f*_0_ during speech perception, yet *how* they achieve this has received little attention in the empirical or theoretical literature. Research on infants’ perception, production, and learning of the functions carried on *f*_0_ has focused mainly on a single specific domain of interest, for example, music, lexical tone, or intonation. To explain how infants learn to perceive the multifaceted and cross-domain *f*_0_ signal, it will be necessary to integrate findings across those different domains of interest. The purpose of this paper is to sketch out an approach to doing that across three *f*_0_ functions: tone, intonation and emotion. We first review empirical studies on infants’ acquisition of the three functions of interest along with their interactions. After that, theoretical considerations are discussed, followed by the proposal of a novel hypothesis.

## Infants’ Acquisition of Tone, Intonation, and Emotion Carried on *f*_0_

### Tone

Around 60–70% of world languages are tonal (Yip, 2002), predominantly using contrastive *f*_0_ variations to differentiate lexical and grammatical changes. Spreading across Asia, Africa, (indigenous) America, Europe and South Pacific regions (Maddieson, 2013), tone languages are spoken by more than half of the world’s population (Fromkin, 2014). Among tones, the predominant *f*_0_ changes lie in pitch height (level, register) and pitch direction (contour, slope; Chao, 1947; Gandour, 1983; Gussenhoven, 2004). Of particular interest are tone languages that rely on lexical tones to distinguish word meanings. For instance, the syllable [ji] in Cantonese means “cure” when bearing a high level tone, but “son” with a low falling tone (Francis et al., 2008). In a tone language such as Mandarin, *f*_0_ carries the primary cues for perception (Gandour, 1983; Massaro et al., 1985; Lee and Lee, 2010), in addition to secondary cues such as intensity and duration (Jongman et al., 2006). The stark difference in *f*_0_ functions in lexical-tone versus non-tone languages raises important questions about how these typological differences influence the development of speech perception, speech production, and word learning.

Speech perception research has shown clear differences in the way that speech is perceived by tone and non-tone language learning infants (Fikkert et al., 2020) as well as by adults (Burnham and Singh, 2018; Liu et al., 2022). Such studies have demonstrated increased tonal sensitivity over the first year after birth for tone language learners and decreased sensitivity for non-tone language learners (Mattock and Burnham, 2006; Mattock et al., 2008; Yeung et al., 2013). However, empirical evidence in the last decade appears to challenge these canonical patterns. For instance, there appears to be an age-based increase in sensitivity to certain tonal contrasts for both tone and non-tone language learning infants (Chen and Kager, 2016; Chen et al., 2017; Tsao, 2017; Ramachers et al., 2018; Singh et al., 2018), and behavioural and neural studies report that bilingual infants tend to be more resilient in perceiving and learning tones even when they do not exist in these infants’ linguistic repertoires (Graf Estes and Hay, 2015; Liu and Kager, 2017a; Liu et al., 2019). Further, a U-shaped sensitivity has been reported in non-tone language learning infants, such that the decline in sensitivity observed over the first year of life is reversed in their second year (Liu and Kager, 2014, 2017a; Götz et al., 2018). Thus, while initial investigations into infant speech perception showed expected declines in sensitivity for tonal contrasts for infants learning a non-tone language, more recent studies suggest that the developmental trajectory requires a more nuanced theoretical interpretation (for similar observations on the development of consonant perception, see Tyler et al., 2014; Liu and Kager, 2015).

Tone production studies typically involve tone language-learning infants, who start producing *f*_0_ contours around 7 months (Chen and Kent, 2009). It is unclear whether the *f*_0_ produced is on a lexical or utterance level (or both), however, because adults cannot identify the ambient language when listening to the babbling of 8–12-month-old English and Mandarin-learning infants extracted from recordings (Lee et al., 2017). Mature production can be observed shortly after 2 years of age (Li and Thompson, 1977; So and Dodd, 1995; Hua and Dodd, 2000; Hua, 2002; To et al., 2013, for a review, see Peng and Chen, 2020). Recent acoustic analyses challenged this conclusion, however, as they have revealed substantial differences between children and adults’ tone production. Mandarin-learning children have been found not to reach an adult level of tonetic realisation until the age of 5 (Wong et al., 2005; Wong, 2012a,b, 2013), possibly due to complex tone articulation (Wong, 2012a) or tonal rules (Chen et al., 2015; Wewalaarachchi and Singh, 2016).

The conflicting findings also extend to word learning. To learn a tone language, children need to associate lexical items that differ minimally in tonal contrasts with different word meanings. Making such associations does not appear to be easy for children at 2–3 years (Shi et al., 2017) and the lexical encoding does not stabilise until around 4–5 years (Singh et al., 2015). Sensitivity to tonal contrast is not required for non-tone language learning infants, yet they are sensitive to *f*_0_ variations on words at 7.5 and 18 months (Singh et al., 2008, 2014). While 14-month-olds are able to associate non-native tones with different objects, that ability decreases at 18 months (Hay et al., 2015; Liu and Kager, 2018). By 2.5 years, they no longer consider *f*_0_ change to be lexically relevant (Quam and Swingley, 2010).

Mixed findings in perception, production, and learning trajectories among tone and non-tone language-learning infants require further investigation. In this paper, we raise the hypothesis that these discrepancies can be attributed to other functional uses of *f*_0_, which are linguistically and paralinguistically relevant in all spoken languages as they can also manifest on the utterance level, such as intonation.

### Intonation

All spoken languages employ intonation (Best, 2019), where *f*_0_ acts at a phrasal level (distinct from the word-level tone, and in addition to other cues such as voice quality; Ladd et al., 1985). When learning a language like English, children need to know that different *f*_0_ contours applied to the same utterance can signal different (e.g., narrative, interrogative) connotations. Intonation can convey linguistic information, facilitate the acquisition of other linguistic components (e.g., words; Thiessen et al., 2005), raise attention (Sullivan and Horowitz, 1983), and carry speakers’ intentions (Gussenhoven, 2002; Esteve-Gibert et al., 2017). Adult listeners can encode both focus and interrogative meaning in intonation (Liu and Xu, 2005). Arguably, this makes intonation a unique component, as it spreads across linguistic and paralinguistic fields and serves grammatical, pragmatic and affective functions (Snow and Balog, 2002). Furthermore, intonation plays a crucial role in caretaker-infant interactions and communications (Stern et al., 1982; Fernald and Simon, 1984; Fernald, 1989).

Infants’ perception and production of intonation develop concurrently with tone throughout infancy and early childhood. Newborns are sensitive to intonation in speech (Nazzi et al., 1998; Sambeth et al., 2008) and 6-month-olds can use pitch contours to parse utterances into clauses (e.g., Seidl, 2007). By 6 and 9 months, European Portuguese-learning infants can discriminate single prosodic-word utterances differing in statement (falling) or yes–no question (falling-rising) intonation (Frota et al., 2014; Frota and Butler, 2018). Despite their sensitivity to the *f*_0_ differences that characterise intonation, children do not appear to rely strongly on intonation to signal conversational turn taking until 3 years and onwards (Keitel et al., 2013). Why they are reluctant to do so at earlier ages needs to be understood.

Arguably, intonation production starts from birth with crying (Mampe et al., 2009) and vocalisation shortly after birth (Kent and Murray, 1982). Newborn infants’ crying patterns already reflect the intonation patterns of their native language (Mampe et al., 2009; Wermke et al., 2016, 2017; Manfredi et al., 2019; Prochnow et al., 2019). Infants begin with a predominant falling pitch contour then progress to other *f*_0_ patterns, with accent range increasing with age (Snow, 2001). The production of pitch register stabilises in the single-word period, and core features are controlled in the two-word stage (Snow and Balog, 2002). However, the development of intonation production in the first 2 years of life is not linear. At the end of the first year after birth, rising and falling contours are produced with a smaller accent range in comparison to the 6–9 and above 18-month-olds. This U-shaped pattern needs further investigation and explanation.

With respect to the interaction between tone and intonation, researchers are prone to argue for a linguistic status of tone and an ambiguous status of intonation: from a categorical perspective, studies favour evidence for discrete tone but not intonation categories, as one “intoneme” may consist of various intonational elements (Tonkova-Yampol’skaya, 1969; but see So and Best, 2014 on “i-category”). Tone-language speakers show distinct tone and intonation processing differences on single-syllable units, not only in the neural organisation of subcortical and cortical structures but also hemispheric lateralisations (Chien et al., 2020), although to date, no consensus has been reached on whether intonation is dominantly processed in the left or right hemisphere. An utterance-final rising *f*_0_ tends to be a universal cue for the perception of interrogation (Gussenhoven and Chen, 2000; Liang and Heuven, 2007), but perception of intonation appears to be tone-dependent. In Mandarin, a yes/no question is more easily identified when the utterance ends with a falling than a rising tone (Yuan, 2011), and a declarative versus interrogative contrast elicits strong mismatch negativity responses on syllables with falling but not rising tones (Ren et al., 2013). Research connecting intonation with word learning is relatively scarce. Although English speakers demonstrate the presence of long-term memory traces for prosodic information in the brain (Zora et al., 2015), English-learning 2-year-olds do not interpret salient pitch contour differences (rising-falling vs. falling-rising) as inherent to novel words (Quam and Swingley, 2010).

Such tone-intonation interaction in perception is not restricted to speakers of a tone language. Among non-tone language speakers, the component that stabilises the earliest, pitch register (Snow and Balog, 2002), can facilitate the perception of non-native tone contrasts (Liu et al., 2022). Non-tone language speakers’ knowledge of intonation also appears to influence tone perception. For instance, the rising versus falling tones in Mandarin Chinese are similar to the declarative versus interrogative *f*_0_ patterns in languages such as English (Braun and Johnson, 2011; So and Best, 2011, 2014). Indeed, when examining American English-learning infants’ Mandarin tone-object association at 14 months, infants were more successful for words with a rising tone than for words with the other three Mandarin tones (Hay et al., 2015, 2019). This suggests that they may have been able to capitalise on their developing sensitivity to English rising pitch intonation for perception of non-native words differing by lexical tone. Adopting intonation patterns from a non-tonal native language for perception of non-native tones is consistent with theories of perceptual assimilation (Best, 1994, 2019; Best et al., 2009; So and Best, 2010, 2014), which may provide a potential theoretical explanation for the U-shaped developmental pattern reported in infant perception of non-native tones (Liu and Kager, 2014, 2017a; Götz et al., 2018). Children learning non-tone languages may become less sensitive to certain *f*_0_ patterns as they recognise that tonal variations do not signal lexical distinctions in their native language, while also learning the complementary functions that *are* carried on *f*_0_.

The nonlinear developmental trajectory for intonation from infancy to toddlerhood (Snow and Balog, 2002), the restricted use of *f*_0_ as a cue in intonation in early childhood (Keitel et al., 2013), and the overlap between tone and intonation in adulthood across the world’s languages (e.g., Gussenhoven and Chen, 2000) all highlight the need to comprehensively understand *f*_0_ functions along the developmental trajectory. Additionally, research on infants’ acquisition of intonation may benefit from considering the prosodic and information structures of intonation, but few studies have taken this approach (Frota and Butler, 2018). For example, according to Autosegmental-Metrical accounts (Pierrehumbert, 1980; Grice et al., 2006; Ladd, 2008; Arvaniti and Fletcher, 2020), intonation is composed of a series of tonal events. To reveal the trajectory and mechanisms infants use to recognise word- and phrase-level prosody from continuous speech, it may be necessary to take the componential structure of intonation into consideration. Further, the visual aspect of intonation, often discussed in sign languages (e.g., Dachkovsky and Sandler, 2009), it still poorly understood in spoken languages. While expressing uncertainty, speakers not only use prosodic cues such as rising intonation, but also facial cues involving eyebrow raising, head tilting, furrowing, etc. (Dijkstra et al., 2006; Roseano et al., 2016).

In the next section, we attempt to explore the *f*_0_ function in the domain of emotion, as well as the entanglement between the intonational and emotional functions in speech directed to infants.

### Emotion

At first glance, there are differences in how theories consider *f*_0_ between linguistic and emotional domains. This is not surprising since emotion theories typically focus on visual emotional signals (e.g., facial expressions) rather than how emotion is coded in speech. Theoretical debates centre on whether humans possess innate basic emotion categories, in both facial expressions (Chong et al., 2003; Gendron et al., 2018) and emotions in vocalisations (Sauter et al., 2010, 2015; Gendron et al., 2014, 2015). Empirical evidence suggests distinct processing of *f*_0_ functions in intonation and in emotion. Emotional voice cues are processed predominantly in the auditory cortical areas in the right hemisphere, whereas phonemic cues are processed mainly in the left (Kotz et al., 2006; Scott and McGettigan, 2013). Limited studies have discussed the interaction between linguistic and emotional *f*_0_ functions (Kotz and Paulmann, 2007; Pell and Kotz, 2011). It is unclear whether certain regions are responsible for *f*_0_ variations in both emotional and linguistic states (Frühholz et al., 2012; Liebenthal et al., 2016).

For preverbal infants, perception of emotion is critical for survival in a social world, as it constitutes one of the critical social cognition skills. While emotion signals in the visual domain are most representative in a speaker’s face and body language, they are carried primarily by *f*_0_ in speech (Remez et al., 1981; Ladd et al., 1985; Scherer, 1986, 2003; Goldbeck et al., 1988). There are also secondary cues for emotion in speech (Murray and Arnott, 1993; Banse and Scherer, 1996; Bänziger et al., 2015; Pell et al., 2015), including intensity and speech rate (Scherer, 1986), pausing structure (Cahn, 1990) and duration (Mozziconacci, 1998), and timbre/voice quality (Gobl et al., 2002; Gobl and Chasaide, 2003; Yanushevskaya et al., 2018). In particular, *f*_0_ modulates and strengthens the affective and motivational contexts in both infants (Stern et al., 1982) and adults (Frick, 1985). It also has an advantage over other cues, such as timbre, that it is simple to measure and quantify.

With respect to emotion perception, infants’ ability to experience and perceive emotion has been hypothesised to develop as a function of neural development, increasing the capacity of processing emotional concepts with the aim of assigning meaning to sensory inputs and guiding behaviour (Hoemann et al., 2019). In their first year of life, infants are sensitive to emotions expressed from different cultures (Liu et al., 2021), and employ different attentional strategies based on their native culture (Geangu et al., 2016). Although emotional *f*_0_ is highly salient in the environment from the beginning of life (ManyBabies Consortium, 2020), and its development is likely linked with the neuro-cognitive development of socio-emotions, the detailed trajectory of emotional *f*_0_ remains unclear.

There appear to be *f*_0_ patterns with distinct acoustic characteristics for different emotions (Liu and Pell, 2012; Wang and Lee, 2015), although findings are mixed on whether emotional *f*_0_ patterns are universal or culturally-specific (Murray and Arnott, 1993; Pell et al., 2009; Li, 2015). Some perception studies have suggested a universal association between high *f*_0_ and positive emotion (e.g., happiness, Ortony et al., 1990; Ilie and Thompson, 2006; Belyk and Brown, 2014), but the same trend has not been observed in other corpus studies (Laukka et al., 2005; Goudbeek and Scherer, 2010). The *f*_0_ acoustics of the same emotional tone can vary across studies in height and range (Pell et al., 2009), along with other cues such as intensity and duration (Wang and Lee, 2015; Wang and Qian, 2018). Furthermore, cross-linguistic and cultural differences have been reported in both the acoustic manifestation (Douglas-Cowie et al., 2003; Anolli et al., 2008; Wang et al., 2018) and the interpretation (Koeda et al., 2013) of *f*_0_. Despite this substantial variation, infants appear to identify regularities to build their knowledge.

There has been a debate in the literature on the processing of emotions in (visual) facial expressions about whether universal categories of basic emotional categories (e.g., happiness, anger) exist (Gendron et al., 2018). Infants can disambiguate between some emotional categories (Caron et al., 1985; Haviland and Lelwica, 1987; Soken and Pick, 1999; for a review, see Widen, 2013), yet it is unclear whether they conceptualise and abstract emotional features such as valence or arousal (Ruba et al., 2020). In comparison, research on processing of (auditory) vocal expressions of emotion is relatively scarce. Unlike 3-month-olds, infants at 5 months can discriminate between vocal expressions of positive and negative valence, but they do so reliably only in the presence of a face (Walker-Andrews and Grolnick, 1983; Walker-Andrews and Lennon, 1991). Infants aged 7 months process emotions of positive and negative valence differently, not only in facial expressions (Nelson and De Haan, 1996) but also in emotional prosody (Grossmann et al., 2005). With respect to the production of emotional *f*_0_, a parental rating study has shown that vocalisations of 2-month-olds can be judged to fit along a comfort-discomfort dimension (Papoušek, 1989). Infants often use prosody, including (high) *f*_0_, to signal what is perceived by their caretakers as emotional cues, be it wailing of fear or crying for attention (for a review, see Bryant, 2021). Little is known about the two-way relationship of *f*_0_ functions in tone and emotion, although language background (tone vs. non-tone languages) has been shown to play a role. Larger *f*_0_ variations of emotional tones are produced by non-tone than tone language speakers (Ross et al., 1986; Anolli et al., 2008; Wang et al., 2018), suggesting that the lexical function of *f*_0_ constrains its use for emotional function.

Some studies have shown that emotional *f*_0_ can facilitate word learning (for a review, see Doan, 2010). For example, words with emotional variations are better recognised in fluent speech by English-learning 7-8-month-olds than words without such variability (Singh, 2008). Infants aged 10.5 months showed significant positive recognition scores for words familiarised in happy but not in neutral emotion text passages (Singh et al., 2004). Words produced with an emotional *f*_0_ assist infants in establishing representations and facilitate their word learning. While this does not automatically imply that they have decoded the emotional function carried on *f*_0_, they are clearly sensitive to the *f*_0_ differences between words produced with a neutral versus emotional *f*_0_. Infants in their first year of life appear to have the capacity to separate linguistic and emotional functions of *f*_0_, but no direct evidence of that has been reported.

Discussion on the interaction between intonational and emotional *f*_0_ functions can be found in the area of infant-directed speech (IDS), a distinctive speech style that caretakers use to communicate with infants (Fernald, 1985, 1992). IDS is more exaggerated, with higher *f*_0_ and wider *f*_0_ ranges than adult-directed speech (ADS). Infants prefer IDS over ADS across the world’s languages (ManyBabies Consortium, 2020). Some identify intonation as the key reason for this preference (Katz et al., 1996), whereas others attribute it to its attention-grabbing qualities (Burnham et al., 2002) and the positive emotion embedded in IDS (Singh et al., 2002). Infants appear to be sensitive to *f*_0_ variations as early as 4 months of age, when they prefer *f*_0_ but not amplitude or duration variations in IDS (Fernald and Kuhl, 1987). The fact that pragmatic functions encompassing both intonation and emotion, such as approval or prohibition, are more clearly expressed in IDS than in ADS, suggests that infants are capable of identifying those *f*_0_ functions (Fernald, 1989; Moore et al., 1997). Indeed, as early as 5 months, infants are able to associate positive emotion in IDS with approval vocalisations, and negative emotion with prohibition vocalisations (Fernald, 1993). The functions of IDS appear to change over the first year of life, with ratings of mothers’ IDS showing general decrease in comforting and soothing functions, and an increase in attentional and directive functions (Kitamura and Burnham, 2003). Infants’ preferences for those functions appear to follow the same developmental trend (Kitamura and Lam, 2009). Despite infants’ clear sensitivity to these *f*_0_ patterns, another study suggests that children do not consider *f*_0_ in speech as a reliable cue to indicate emotions until around 4–5 years of age (Quam and Swingley, 2012).

To our knowledge, no study has attempted to tease apart the three-way interaction between tone, intonation, and emotional functions in *f*_0_. Trends may be observed in emotional *f*_0_ from its immense variations, but not “rules” in the same sense as tone (e.g., “a tone language has a set of fixed pitch variations”) or intonation (e.g., “a question usually has a rising pitch”). Thus, while there are broad indicators about the association between *f*_0_ and emotion, this relationship, as well as its consistency across languages and cultures, is still under investigation. The interactions in between tone, intonation and emotion remain unclear, and research on IDS cannot efficiently disentangle its impact from intonational or emotional perspectives.

### Summary

The fluctuating *f*_0_ signal contains overlapping information from different sources that infants need to decompose and recognise. We have focused on three distinct functions carried by *f*_0_; tone, intonation, and emotion. It is not yet clear whether languages differ from each other in the way that emotion is expressed using *f*_0_, but there are clear differences in the ways that languages use *f*_0_ for tone and intonation. Infants do not know innately whether the information in *f*_0_ refers to tone, intonation, or emotion. They must learn which aspects of the fluctuating *f*_0_ signal correspond to different functions.

Studies on the developmental trajectories of infants’ sensitivity to the tonal, intonational, and emotion aspects carried on *f*_0_ have yielded mixed findings. Unstable and fluctuating developmental trajectories have been reported for tone, not only for infants learning a tone language but also for those learning a non-tone language in the first 2 years of life. Similarly, infants’ intonation development does not appear to be linear before Year 2, and children do not use *f*_0_ for intonation reliably until after Year 3. Although the contribution of *f*_0_ on emotion is widely acknowledged, incongruent findings have been reported across the world’s languages. Reliable use of *f*_0_ as a cue to indicate emotion has only been found after Year 4 (Quam and Swingley, 2012).

Research on infant speech perception has only recently begun to focus on *f*_0_ and there is certainly more work that needs to be done to establish clear developmental patterns. Nevertheless, it is clear that infants are sensitive to *f*_0_ across domains, in tone (Liu and Kager, 2014), intonation (Frota et al., 2014) and emotion (Singh et al., 2004), and it appears that robust knowledge about tone is learned ahead of intonation and emotion. This observation is consistent with the idea that discrete categories for tone seem to be established earlier and more easily than they are for intonation (Tonkova-Yampol'skaya, 1969; Snow, 2006; Yeung et al., 2013). Indeed, it could be argued that the variability in the way that the three functions are represented in *f*_0_ increases from tone, to intonation, then emotion. Such variability would make an infant’s job of learning the *f*_0_ patterns even more challenging, which may explain the developmental progression and fluctuation across domains.

Although traces of overlap in between these domains appear in literature, there is insufficient empirical data to disentangle the interactions between tone, intonation and emotion in the development of *f*_0_ perception. To arrive at a clear explanation of how infants learn to use *f*_0_ cues in linguistic and paralinguistic functions, it is necessary to formulate a theoretical framework that incorporates *f*_0_ functions across multiple domains.

## Theoretical Considerations

Investigating how infants solve the puzzle of decomposing *f*_0_ into different functions is a rare opportunity to observe language development across different communicative domains. One interesting aspect of *f*_0_, from a developmental perspective, is that an *f*_0_ pattern that signals a tonal function in one language could be perceived as intonation in another. Proposing a perspective that can conceptually integrate across all three lines of inquiry – tone, intonation and emotion – may seem ambitious, but it is necessary to consider all of these aspects to understand how infants learn to decode *f*_0_. Given the developmental patterns that have been observed for the three domains, a purely bottom-up statistical learning solution seems unlikely. Rather, infants may require multimodal experiences from their environment to develop functional speech communication skills. Our current understanding of how tone or intonation is coded in the visual modality, and how emotion is coded in the auditory speech signal is rudimentary. Nevertheless, addressing the multifunctionality of the speech signal using a global approach, conveying linguistic, paralinguistic, and affective information simultaneously, is critical for a comprehensive model of speech development. Any theory addressing *f*_0_ perception and development will need to be able to explain how children acquire their native *f*_0_ functions and account for the mixed findings observed in previous literature. On these bases, we argue for four critical aspects that must be properly addressed by any theories concerning *f*_0_ perception and development.

- *Disambiguation*: how infants disentangle and recognise multiple overlapping *f*_0_ patterns
- *Categorisation*: how infants learn that those patterns correspond to a given (native) linguistic or paralinguistic function
- *Accomodation*: how infants tackle *f*_0_ functions that deviate from their native functional use
- *Interaction*: why recognition, learning and cue weighting of *f*_0_ fluctuate along the development

Below, we consider how developmental theories of speech perception, cognition, and statistical learning may contribute to a broad theoretical approach to explaining the eventual successful acquisition of *f*_0_ functions.

### Speech Perception

From a developmental perspective, *Perceptual Attunement* accounts (Werker and Hensch, 2015; Reh et al., 2020) propose that an infant’s perception gradually shifts from universal into native or environmentally-attenuated perception patterns. Such changes occur across domains and modalities, fitting well in the aspect of *categorisation*. Such accounts associate well with, and arguably, lay the foundation of speech processing theories. For linguistic functions such as tone and intonation, infants typically exhibit initial biases or universal sensitivity, and quickly tune into the *f*_0_ patterns of their native language (Burnham, 1986). Meanwhile, assimilations or perceptual difficulties surface since non-native or unfamiliar *f*_0_ patterns are tuned out. Having said that, discrepancies from the attunement process have been reported for native and non-native *f*_0_ patterns (Fikkert et al., 2020). Though overlapping *f*_0_ patterns have been used as a possible explanation for these findings, theories of perceptual attunement will need to demonstrate *disambiguation*: how infants overcome overlaps in (e.g., *f*_0_) functions along the developmental trajectory.

Further, models and theories of infants’ acquisition of their L1 phonological system have been devised to explain how infants tune in to the phonetic features that signal phonological similarities and differences in the language of their environment (e.g., Best, 1994; Escudero, 2005; Kuhl et al., 2008; Polka and Bohn, 2011). The focus of these models has been on the acquisition of consonants and vowels (henceforth, *phones*). Here, we use the framework of the *Perceptual Assimilation Model* (PAM; Best, 1994; Best et al., 2009, Tyler et al., 2014) to consider how such models might account for the acquisition of *f*_0_ functions.

A key empirical observation that led to the development of PAM was that English infants and adults had high discrimination accuracy for non-native Zulu click consonants despite never having encountered them before (Best et al., 1988). When asked to write down what they heard, all participants reported relying on non-speech characteristics of the consonants (e.g., water dripping, fingers snapping, or tongue popping). To account for this, PAM proposes that non-native phones may be perceived as speech (i.e., assimilated to the native phonological system) or as non-speech. When perceived as speech, a non-native phone may be assimilated as categorised (as a good, medium, or poor exemplar of a native phonological category) or uncategorised (not a clear exemplar of any single L1 category). Discrimination of non-native phonemes that are perceived as speech is crucially dependent on how it is assimilated to the native phonological system. Sometimes natively tuned perception will support discrimination (e.g., when each non-native phone is assimilated to a different L1 phonological category) and sometimes it will make it difficult to perceive any differences between them (e.g., when the non-native phones are perceived as equally good or poor exemplars of the same L1 category). Contrasting non-native phones that are perceived as non-speech (e.g., click consonants) are discriminated well by adults because they learned that the phonetic features of these categories are not used for linguistic purposes in their native language. Consistent with this account, native speakers of the click languages Zulu and Sesothu predominantly perceived non-native!Xóõ click consonants as speech (Best et al., 2003). Both click consonants in one of the !Xóõ contrasts were perceived as the same L1 click consonant category by both Zulu and Sesothu listeners. Importantly, English listeners perceived the same click consonants predominantly as non-speech and their discrimination of the contrast was more accurate than both groups of click language speakers. It appears that the English speaking adults had learned, as infants, that the phonetic characteristics that correspond to click consonants were not part of the L1 phonological space.

According to PAM, infants transition from language-independent phonetic sensitivity to natively tuned perception by recognising higher order invariant information in articulatory patterns through processes of perceptual learning (Gibson and Pick, 2000). Phonetic variability is crucial for phonological development because infants need to learn not only those phonetic differences that signal a difference in meaning (the principle of phonological distinctiveness), but also those variable phonetic characteristics that define a category (the principle of phonological constancy; Best et al., 2009; Best, 2015). The region of phonetic space that is dedicated to speech is known as the phonological space. Click consonants would fall outside of the phonological space for English speakers but they would fall inside the phonological space for click language speakers. The development of phonological categories is beneficial for L1 perception because it supports accurate and rapid detection of the critical phonetic differences that signal a potential difference in word meaning. However, once infants have begun to tune into the L1 phonology, non-native speech is also perceived in terms of its similarities and differences to their developing L1 phonological categories. If they happen to perceive each phoneme in a non-native contrast as different L1 phonological category (e.g., one phoneme as /b/and the other as/d/, a PAM two-category assimilation) then their natively tuned perception will still support rapid and accurate discrimination. However, if both non-native phonemes are perceived as the same L1 category (e.g., the Hindi dental vs. retroflex plosive contrast for English native speakers, Werker and Logan, 1985; a PAM single-category assimilation), then discrimination is poor.

If fluctuating *f*_0_ patterns were considered in a similar way as the varying articulatory-acoustic patterns that demarcate consonants and vowels, then it is conceivable that infants might use similar learning mechanisms to separate the linguistic, paralinguistic, or extralinguistic functions carried on *f*_0_. For example, the *f*_0_ patterns that are used in a tone language for lexical distinctions may be similar to those used for other functions in a non-tone language, such as intonation (for a discussion, see, Best, 2019). The developmental changes in infants’ responses to *f*_0_ fluctuation might then be explained by infants’ learning and recognition of the various functions at different ages. For infants who experience phonological characteristics of a non-tone language, *f*_0_ is irrelevant for lexical distinctions. This may explain why discrimination of tonal contrasts initially declines. The subsequent improvement would then be due to the development of sensitivity to other types of *f*_0_ information. Thus, from the perspective of the Perceptual Assimilation Model, *disambiguation* and *categorisation* occur through processes of perceptual learning. *Accommodation* may be observed if infants perceive a non-native *f*_0_ pattern as consistent with a different type of function in their L1, and *interaction* may be explained by the different timescales for perceptual development of linguistic, paralinguistic, and extralinguistic information.

### Cognition

Another potential joinder of the three areas of *f*_0_ functions resides in cognitive competition. Theories such as the *Functional Load Hypothesis* (FLH, Berinstein, 1979) postulate that our prosodic space of a given language is finite, and therefore, assume competition in phonological processing. Under FLH, it would be more cognitively demanding to process *f*_0_ contours that simultaneously carry more than one type of function.

The FLH predictions provide indirect explanations for *disambiguation,* as presumably, competition across diverse *f*_0_ functions may facilitate their recognition, disentanglement and establishment of *f*_0_ categories. These predictions also offer viable ways of empirically examining FLH as a hypothesis. Having said that, existing findings are mixed (van Heuven, 2018). FLH is supported by studies investigating parameters competing within the prosodic domain. Supported by phonological and acoustic analyses, Remijsen (2002) has shown that it is highly unlikely for a tone language to feature lexical stress because that would create competition (and thus ambiguity) between the pragmatic and the lexical functions of *f*_0_. Using phonological and acoustic analyses, Remijsen (2002) concluded that it is implausible for lexical tone and lexically contrastive stress accent to co-exist in the word-prosodic system of a language. Nevertheless, challenges appear to lie in *interaction*: FLH would need to explain how parameters from different domains within phonology (e.g., prosodic vs. segmental domains) and beyond (e.g., linguistic vs. paralinguistic domains) compete against one another. In other words, it is unclear whether and to what extent information across domains and modalities fights for cognitive resources during processing. FLH concentrates on the linguistic domain and the emotional aspect has not been directly considered (although it was alluded to in Chen, 2005). Nevertheless, the FLH postulation seems to imply that languages encoding *f*_0_ in both tone and intonation would have less functional space left to encode *f*_0_ in emotions. Note that caregivers may assist, consciously or unconsciously, in the reduction of functional loads in the course of infants’ learning. For instance, they may package messages in IDS to reduce processing challenges for certain *f*_0_ functions.

The FLH faces challenges incorporating cross-domain or cross-modal facilitation effects. That is, information perceived in one domain (e.g., vision) may support perception and learning of information in another domain (e.g., speech). These are often referred to as bootstrapping or anchoring effects. For instance, the prosodic bootstrapping hypothesis suggests that infants may use prosodic information to discover utterance and word boundaries (Seidl and Johnson, 2006; Johnson et al., 2014), and knowledge of word semantics may further cue syntactic categories (Höhle, 2009). Along the same lines, various sources of information from the ambient environment provide anchors to facilitate children’s *f*_0_ *disambiguation* and *categorisation* along the developmental trajectory. The command of one *f*_0_ function may facilitate another even when they are simultaneously presented. FLH, or any cognitive model, will need to clearly explain the degree of interaction between competition and facilitation in co-occurring functions.

What has not been discussed, but links closely with the FLH mechanisms, is how infants cope with cognitive demands and how increased neurocognitive ability affects children’s perception and learning. It takes children years to master linguistic and pragmatic functions. Taking *Theory of Mind* (ToM) as an example, ToM refers to the understanding of distinctions between individuals’ mental states, mental constructs, physical entities and their overt actions (Gopnik and Wellman, 1992; Wellman, 1992). ToM is crucial for children’s socio-emotional development. What needs to be explored is how children’s gross and specific (e.g., socio-emotions) cognitive development attributes to the learning of emotional *f*_0_.

### Statistical Learning

Statistical learning refers to the ability to acquire information solely based on relevant statistical distributions in the ambient environment, and *Statistical Learning* accounts argue that infants utilise their innate statistical (Saffran and Kirkham, 2018) and relational (Ferry et al., 2015) learning ability to acquire new information. For instance, 8-month-olds are able to segment words from fluent speech based on one and only one cue: the statistical relationships between neighbouring syllables (Saffran et al., 1996; but see Johnson and Tyler, 2010).

While statistical learning accounts have been used to describe acquisition of a single *f*_0_ function (e.g., lexical tone, Liu and Kager, 2017b), its explanatory power faces evident challenges in *disambiguation* and *categorisation*. A purely bottom-up learning of a statistical distribution does not appear sufficient to explain *disambiguation* if *f*_0_ is the only statistical distribution available. By comparison, vowels may be disambiguated on the basis of multiple information sources (e.g., the first, second, and third formants, and duration). Even though *f*_0_ serves as the primary acoustic correlate of emotional tones (Scherer, 2003), its usage differs between tone and non-tone language speakers, with greater *f*_0_ variations in the productions of the latter group. It seems likely that statistical learning of *f*_0_ patterns would require correlated statistical distributions from other information sources. This may include phonation type (e.g., creaky voice) or tone-vowel interactions (Shaw and Tyler, 2020) for tone, and voice quality for both intonation (Ladd et al., 1985) and emotion (Yanushevskaya et al., 2018). Cue-weighting, or differences in listeners’ weighting of acoustic cues (e.g., between *f*_0_ and secondary cues such as amplitude and duration, Ross et al., 1986), likely further modulates statistical learning.

With respect to *categorisation*, statistical learning ability does not appear to be constant across ages. Its efficacy changes dynamically over a child’s development. However, the direction of such change, or the statistical learning efficacy across ages, is currently a matter of debate. On the one hand, a meta-analysis has reported increased effect sizes with age in the first year of life (Cristia, 2018), suggesting that older infants are increasingly sensitive to this learning mechanism. On the other hand, behavioural (Yoshida et al., 2010) and neural (Wanrooij et al., 2014) evidence has shown that this learning mechanism may be maturationally delimited, along the perceptual attunement trajectory during which phonetic perception is refined (Liu and Kager, 2017b; Reh et al., 2021). The latter evidence suggests that the learning of sound frequency distributions become increasingly resistant as children grow. Discrepancies in literature have been explained by the different perceptual attunement time windows of speech sounds differing in phonetic representations, space and perceptual/acoustic salience (Werker and Hensch, 2015; Reh et al., 2021). Hence, statistical learning of speech sounds may be at its peak of efficacy during perceptual attunement, when infants’ perception exhibits enhanced sensitivity to input from the environment.

Although the learning mechanism is considered domain- (and even species-) general, individual studies and models typically investigate statistical learning in a domain-specific fashion. Despite the challenge in *disambiguation* and the debate in *categorisation*, in order to achieve learning of diverse *f*_0_ functions, models of statistical learning would require additional focus on the *interaction* mechanisms, with modelling of certain (e.g., *f*_0_) statistical distributions across domains.

### Summary

Similar to the lack of empirical research in studying the interaction of distinct linguistic and paralinguistic functions carried on *f*_0_, none of the existing models and hypotheses seems sufficient in addressing how different *f*_0_ functions disassociate in sensory and cognitive processes, or the extent to which they are processed simultaneously or separately. A theoretical account is required for how infants manage to decompose these overlapping *f*_0_ functions while taking into consideration the differences between these functions across languages/cultures, as well as information integration across modalities.

As summarised in the beginning of this section, to achieve successful learning, infants must rely on fundamental aspects (disambiguation, categorisation, accommodation and interaction). These aspects point out directions where the exploration of diverse *f*_0_ functions may converge. These directions are crucial for us to understand how infants resolve puzzles identified in the literature:

- *Neuro-cognitive Development*, which reflects age-related developmental and maturational changes
- *Environmental Information*, where learning of language and social-emotions from the ambient resources occur
- *Competition and Facilitation*, within and across perceptual and/or cognitive spaces and modalities (e.g., auditory, visual) where information gathers and integrates

Infants eventually sort out their native linguistic and socio-emotional functions carried on *f*_0_. Thus, developmental and environmental aspects such as age and experience will need to be considered when exploring *f*_0_ functions, in line with the first two directions. With respect to category learning, future research should focus on the establishment of *f*_0_ categories for tone, intonation, and emotion. Further that, the degree of flexibility and assimilation when facing a novel/non-native category will need to be explored. Regarding bootstrapping, a theoretical basis will require that infants effectively integrate environmental sources of information and existing knowledge to recognise and disambiguate *f*_0_ functions.^1^ A multimodal view into the issue is also consistent with an ecological approach to perceptual learning and development (e.g., Gibson and Pick, 2000).

To summarise across the four directions, future research should concentrate on how infants decompose and acquire linguistic and paralinguistic functions carried on *f*_0_; to what extent reinforcement or interference may occur with infants’ perception and learning of *f*_0_ functions; and how infants employ environmental resources to disambiguate these functions.

### Our Hypothesis

Considering the gap in discussion of *f*_0_ functions across linguistic and socio-emotional domains, the four aspects concerning *f*_0_ perception and development, and the four directions essential to achieve its functional learning, we propose a *Learnability Hypothesis* that infants require multimodal environmental experiences to decompose and acquire overlapping linguistic, paralinguistic, and extralinguistic *f*_0_ functions. Its predictions are as follows: When faced with *f*_0_ contours carrying multiple functions, perception and learning of a certain function should be enhanced if other functions are not ambiguous, and should be affected if other functions have not been properly learned or cannot be properly identified. Moreover, infants use acquired, environmental and multi-modal cues to anchor and facilitate learning whenever possible.

A representative and measurable case of the learnability hypothesis can be viewed as the “synergy scenario.” For example, infants can use visual cues to disambiguate and decompose different auditory *f*_0_ functions. Congruent audiovisual cues of the same function will lead to corresponding enhancements as well as reduced sensitivity to others. In contrast, incongruent cues may capture infants’ attention, as is the case for deviants against standards in an oddball paradigm in electroencephalogram, or regained attention to new information in a behavioural habituation paradigm. These predictions provide us with viable ways of testing the hypothesis.

One way to examine this scenario would be to use an experimental paradigm that reflects the real world lives and interactions that infants experience, such as using stimuli that mirror real communications that occur in infant-caregiver interactions. Following an associative learning paradigm (Hay et al., 2015; Liu and Kager, 2018), infants’ ability to associate novel objects with an instructor’s *f*_0_s that represent tones could be measured with or without the instructor’s visual intonational and emotional information. Here, the *f*_0_s could be ambiguous, not only reflecting tonal but also intonational or emotional *f*_0_ that are relevant in infants’ native environment. In this case, when the presented visual information matched intonational or emotional *f*_0_, infants should show a reduction in associative learning.

## Conclusion

A diverse array of linguistic and paralinguistic functions are carried simultaneously on *f*_0_. Patterns of *f*_0_ variability differ across languages, such that an *f*_0_ pattern that serves a particular function in one language may serve a different function in another. Adults use native *f*_0_ functions effortlessly, but how infants acquire them remains a mystery. Infants’ unstable learning trajectories raise important questions. For instance, when they no longer treat *f*_0_ differences as potential signals to a change in a certain function, is it due to an insensitivity to *f*_0_ features or due to those features being used for a different communicative purpose? Do infants adopt top-down or bottom-up processing when disambiguating different functions carried on the same *f*_0_? These questions surface from the mixed findings in the literature, across tone, intonation, and emotional domains.

It is important to seek answers to these questions and solutions to the discrepancies observed in the literature. The body of literature needs to be expanded to include infants from a broader range of language environments so that we can understand the course of acquisition. Obtaining the answers through a theoretical and empirical approach, such as the research ideas spawned by our *Learnability Hypothesis*, will improve and integrate theories across research fields, especially when existing models do not appear sufficiently inclusive to address the learning process.

The early years of life lay solid foundations for child learning, assisting our young learners to navigate through the complexities of our modern world. The understanding of how children command the multiple *f*_0_ functions using an ecological approach will function as a benchmark guiding pitch learning in the natural environment; help with the identification of speech or cognitive impairments; better support typical child development; and contribute to multilingual/vulnerable language learning, second/foreign language learning, as well as learning across the lifespan.

## Author Contributions

LL and MT drafted and revised the manuscript. AG and PL revised the manuscript. All authors contributed to the article and approved the submitted version.

## Funding

LL’s writing was partially supported by the European Union’s Horizon 2020 research and innovation programme under the Marie Skłodowska-Curie grant agreement No. 798658 hosted by Center for Multilingualism across the Lifespan at the University of Oslo, financed by Research Council of Norway through its Centers of Excellence funding scheme grant agreement No. 223265. The open access publication fee was supported by Western Sydney University.

## Conflict of Interest

The authors declare that the research was conducted in the absence of any commercial or financial relationships that could be construed as a potential conflict of interest.

## Publisher’s Note

All claims expressed in this article are solely those of the authors and do not necessarily represent those of their affiliated organizations, or those of the publisher, the editors and the reviewers. Any product that may be evaluated in this article, or claim that may be made by its manufacturer, is not guaranteed or endorsed by the publisher.

## Footnotes

^1^Recent evidence suggests that there may be information about *f*_0_ in the face and in head movements that can be used to discriminate lexical tone contrasts (Burnham et al., 2022), but it is not clear from these findings whether such auditory visual speech information would be useful for disambiguating different *f*_0_ functions. Here we consider the role of non-speech environmental information on the acquisition of *f*_0_ functions.
